# Transcriptome data of cultivated tetraploid and hexaploid wheat variety during grain development

**DOI:** 10.1016/j.dib.2018.12.058

**Published:** 2018-12-21

**Authors:** Pranab Kumar Mandal, Shubham Rai, Megha Kaushik, Subodh Kumar Sinha, Rajesh Kumar Gupta, Anju Mahendru

**Affiliations:** a Indian Council of Agricultural Research – National Research Centre on Plant Biotechnology (ICAR-NRCPB), LBS Building, Pusa Campus, New Delhi 110012, India; b Indian Agriculture Research Institute – Division of Genetics, Pusa Campus, New Delhi 110012, India

**Keywords:** Hexaploid bread wheat, Tetraploid durum wheat, Transcriptome, Grain development

## Abstract

Wheat is a major food crop and an important component of human diet throughout the world. There are two major types of cultivated wheat; one is tetraploid durum (pasta) wheat and another one is hexaploid bread wheat. Wheat grain is the reservoir of two major dietary components – carbohydrate and protein, which get accumulated during seed maturation and directly affects yield and quality. Hexaploid, having 6 copies of each chromosome differs to a great extent from tetraploid having 4 copies of each chromosome. Studying the gene expression pattern in developing grain would help in understanding the difference in metabolic process as well as involvement of the genes in these two types of wheat. A transcriptional comparison of developing grains was carried out between the two wheat genotypes; tetraploid (AABB:PDW233) and hexaploid (AABBDD:PBW343) using RNA-seq. Approximately 194 million raw reads were obtained from both libraries. After removal of contaminations, a huge proportion (>99%), of high quality reads were obtained, were aligned to reference genome. A total of 2324 up-regulated and 522 down-regulated genes were identified as differentially expressed between PDW233 vs PBW343. Gene ontology annotation and enrichment analysis gave further information about differentially expressed genes between durum and bread wheat. This information will help in understanding process grain reserve in tetraploid and hexaploid wheat in relation to their nutritional quality.

**Specifications table**

**Table:** 

Subject area	Biology
More specific subject area	Transcriptomics
Type of data	Transcriptome sequences
How data was acquired	Illumina HiSeq. 2000
Data format	Raw data (FASTQ)
Experimental factors	RNA was isolated during grain development
Experimental features	Datasets for hexaploid bread wheat and tetraploid durum wheat
Sample source location	New Delhi, India
Data accessibility	http://www.ncbi.nlm.nih.gov/bioproject/503549

**Value of the data**

• This is transcriptome analysis of two wheat genotypes i.e., tetraploid durum wheat (AABB: PDW233) and hexaploid bread wheat (AABBDD: PBW343) using RNA-seq. These are the most prominent and versatile varieties in North Western Plains of India. Owing to their bread and pasta making qualities, these genotypes were selected for transcriptome analysis during grain filling stages. Our analysis highlighted the genes involved in starch and protein synthesis as well as in stress defense, which are specific for grain development.
• The present study will provide a better understanding on molecular mechanism of accumulation of nutritive reserves in wheat endosperm, which can provide energy for seed germination.
• The data can be a reference transcriptome for various other wheat cultivars of tetraploid and hexaploid wheat and useful for comparative analysis with other progenitors of wheat.

## 1 Data

This article reports the transcriptome data of tetraploid wheat and hexaploid wheat. We obtained more than 192 million clean reads that passed the quality filters (Table 1). The processed reads were used for reference based pair-wise alignment. The overall alignment summary is shown in Table 1 below.

**Table 1:** Read alignment summary of both transcriptomes of the tetraploid and hexaploid wheat.

Genotype name	Raw reads	Clean reads	Aligned read count	Aligned %	Unaligned read count	Unaligned %
Tetraploid (AABB:PDW233)	117,464,834	117,222,916	106,431,419	90.79	10,791,497	9.21
Hexaploid (AABBDD:PBW343)	75,662,740	75,577,214	58,162,696	76.96	17,414,518	23.04

**Fig. 1:**
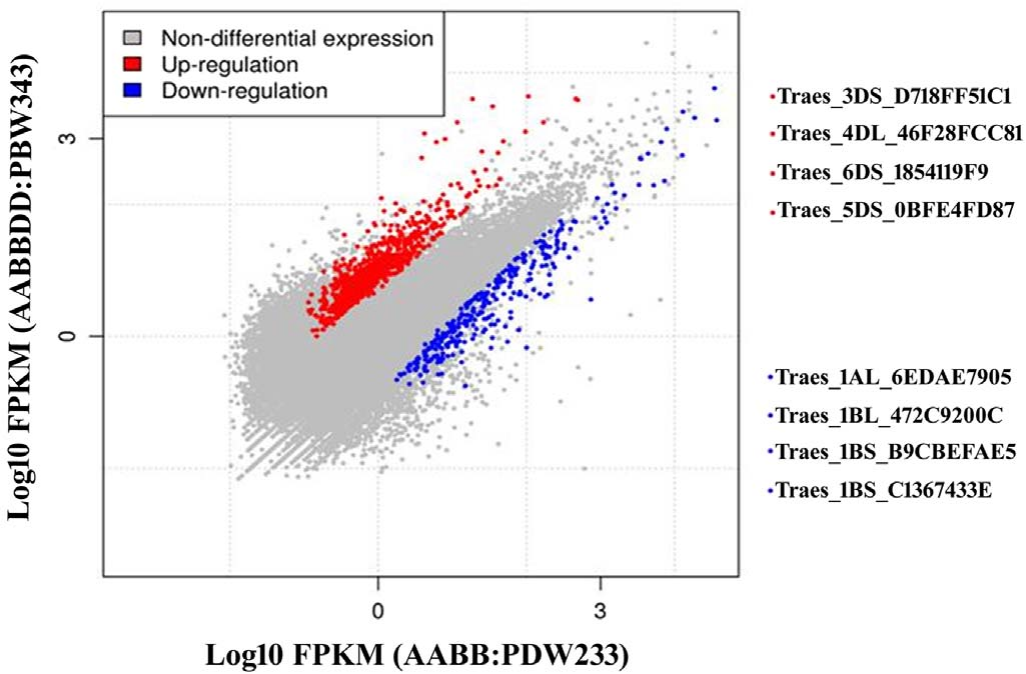
Differentially expressed transcripts between (AABB: PDW233) vs (AABBDD: PBW343).

**Fig. 2:**
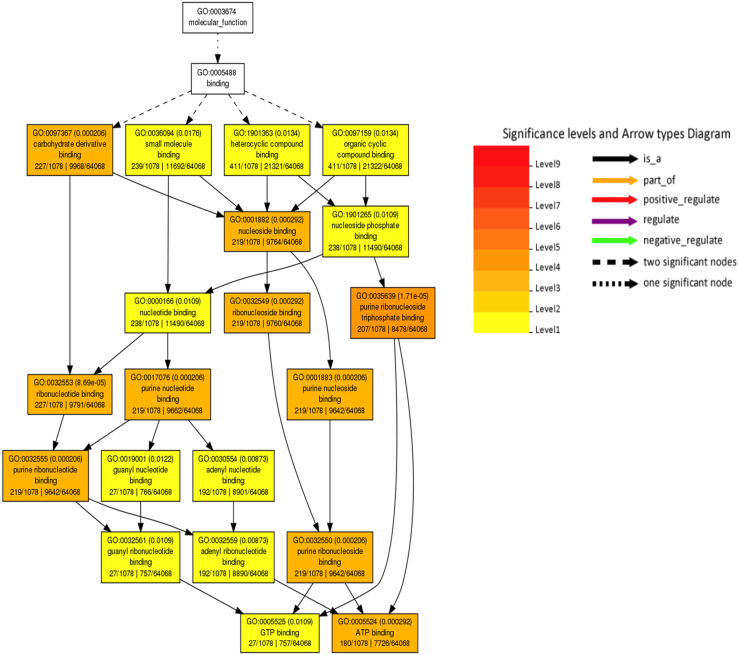
GO enrichment analyses of transcripts found in (AABB:PDW233) vs (AABBDD:PBW343) Agri GO analysis (red higher, yellow lower). The statistically most significant GO terms are given in the figure. Full data sets are available for it in Supplementary Table S2.

Transcriptome analysis of developing grain was compared between two wheat genotypes (PDW233 and PBW343). High quality reads were aligned to ~77% in hexaploid (AABBDD) and to ~91% of tetraploid with the reference genome using Tophat. The value we received is quite acceptable range when compared with previously reported mapping rates [4] and we were able to detect the expression of most of the gene models. A total of 2324 up-regulated and 522 down-regulated genes were identified as differentially expressed between PDW233 vs PBW343 (Fig. 1) (Supplementary Table S1) (Table 2). Using gene ontology, 1078 GO terms were classified to molecular function category, 674 GO terms cellular component category, 616 GO terms were classified for biological process category (Supplementary Table S2). Among these, a large number of genes involved in starch synthesis were detected in our results. Starch synthesis genes were found to be up-regulated in (PBW343), which is the key enzymes in the process of starch synthesis. We found that the genes encoding sucrose synthase (SUSY) [3] which converts sucrose into fructose and glucose were up-regulated with more than +2.9 to 5.7-log_2_-fold in hexaploid wheat compared with AABB genotypes during starch accumulation. Genes involved in the biological process of response to stress, polysaccharide metabolism, multi-organism processes, and the biological function of nutrient reservoir and hydrolase activities were up-regulated in hexaploid wheat compared with AABB genotypes. In the present study, we also identified several important enzyme families, such as peroxidases, (Peroxidases play many roles in plant seeds, such as removing noxious H2O2, facilitating seed maturation by inhibiting IAA activity, and promoting ethylene synthesis) methyltransferases (methyltransferases are responsible for amino transfer, which plays an important role in amino acid synthesis and protein synthesis). Up-regulation of all of these enzymes are critical for successful seed development. Superoxide dismutase is the defense protein showed of +3.98-log_2_ fold up-regulation in hexaploid wheat (AABBDD) compared to tetraploid wheat (Fig. 2).

**Table 2:** Total up and down regulated genes (*p*-value ≤ 0.05) found using Cuffdiff analysis.

(AABB:PDW233) vs (AABBDD:PBW343)	Up-regulated	Down-regulated
	2324	522

## 2 Experimental design, materials and methods

### 2.1 Plant material and growing condition

Wheat seeds of two genotypes were procured from IARI (Indian institute of Agriculture Research, New Delhi, India). Sowing is done on 3rd week of November 2014 at ICAR-NRCPB Net house (28°64″N, 77°16E). Uniform size seeds of each wheat genotypes (tetraploid and hexaploid) were surface sterilized using 0.5% HgCl_2_ and were sown in large size pots (18 in. diameter and 24 in. height) with standard package of practices. Each genotype was grown with three replications, developing grain samples were collected 2 weeks after anthesis (WAA), 3WAA, 4WAA and 5WAA. Grain samples were snap freezed in liquid nitrogen and stored in −80 °C for further use.

### 2.2 RNA extraction, cDNA library construction and sequencing

Frozen grain samples of three biological replicates were pooled. Each pooled sample was ground in and total RNA was extracted using TRIzol^®^Reagent according to standard protocol. The quality of total RNA was checked by electrophoresis and the concentration of total RNA was determined by NanoDrop^TM^ ND 1000 spectrophotometer. RNA with an RNA integrity number (RIN) for PBW343 of 7.6 and PDW233 of 6.1 was only considered mRNA purification. Oligo dT beads (Illumina® TruSeq® RNA Sample Preparation Kit v2) were used to purify mRNA from one microgram of total RNA. Elevated temperature (90 °C) in presence of divalent cations was used to achieve the fragmentation of the purified mRNA. cDNA synthesis was done using random hexamers with Superscript II Reverse Transcriptase (Invitrogen Life Technologies). Agencourt Ampure XP SPRI beads (Beckman-Coulter) were used to clean the cDNA. Illumina adapters were ligated to the cDNA molecules after end repair and the addition of an ‘A’ base followed by SPRI clean-up. The resultant cDNA library was amplified using PCR for the enrichment of adapter-ligated fragments, quantified using a Nanodrop spectrophotometer (Thermo Scientific) and validated for quality with a Bioanalyzer (Agilent Technologies). The libraries were then sequenced on Illumina Hiseq. 2000 platform at SciGenom Next-Gen sequencing facility, Cochin, India.

### 2.3 Sequence data assembly and analysis

The paired ends reads generated were first subjected to quality check In-house Perl scripts and picard tools (version 1.100) was used to remove low quality reads (Phred score < 30). Tophat (version 2.1.0, https://ccb.jhu.edu/software/tophat/) Cufflinks (version 2.2.1;http://cufflinks.cbcb.umd.edu/) softwares were used for reference based assembly [1]. The trimmed sequences were aligned to the Wheat genome and gene model downloaded from Ensembl Plants (ftp://ftp.ensemblgenomes.org/pub/plants/release-29/fasta/triticum_aestivum/dna/). After aligning the reads with reference gene model, the aligned reads were used for estimating expression of the genes and transcripts using cuffinks program (version 2.2.1). Differential expression analysis was carried out using cuffdiff program of cuffinks package. Differential expression analysis was carried out using cuffdiff program of cuffinks package. The regulation for each transcript was assigned based on log2fold change. The transcripts with log2fold change ≥1 (for up-regulated transcripts), ≤−1 (for down-regulated transcripts) with p-value cutoff of ≤0.05 were considered as significantly expressed transcripts.

### 2.4 Functional annotation and biological classification of transcripts

Predicted transcripts were annotated by using blast2go pipeline on default settings. BLASTP were performed with an *E*-value of 1e−5 to align against NCBI non-redundant (nr) protein database for homology search. We used AgriGO (http://bioinfo.cau.edu.cn/agriGO/analysis.php) a web based tool for enrichment analysis. AgriGO uses Singular Enrichment analysis (SEA) method for Gene enrichment following AgriGO SEA parameter settings: fisher test, with yekutieli (FDR under dependency), 0.05 significance level, 5 minimum mapping entries and complete GO and molecular function gene ontology [2].
